# Deposition of NiAl/Al_3_Ni_2_(CrB_2_) Coatings from Ni, Al and CrB_2_ Powders Using Mechanical Synthesis in Planetary Ball Mill

**DOI:** 10.3390/ma17020492

**Published:** 2024-01-19

**Authors:** Maciej Szlezynger, Daniel Toboła, Jerzy Morgiel

**Affiliations:** 1 Institute of Metallurgy and Materials Science, Polish Academy of Sciences, Reymonta 25 Str., 30-059 Krakow, Poland; m.szlezynger@imim.pl; 2 Łukasiewicz Research Network—Krakow Institute of Technology, Zakopianska 73 Str., 30-418 Krakow, Poland; daniel.tobola@kit.lukasiewicz.gov.pl

**Keywords:** coatings, NiAl/Al_3_Ni_2_(CrB_2_), cold-welding, ball mill, hardness, wear

## Abstract

Interest in composite thick coatings with an intermetallic matrix stimulates the development of new deposition techniques like the co-milling of pre-alloyed NiAl powder with platelet-shaped substrates. Obtained coatings were up to several micrometers thick as cold-welding of intermetallic particles was effective only at the start of this process, while later, chipping prevailed over added material. The present experiment covered the co-milling in the planetary ball mill of Ni and Al elemental powders (1:1 molar ratio) with AISI 304 steel platelets for 32 h at 300 rpm. Next, this process was repeated with an admixture of 15 wt.% of CrB_2_ powder. In both cases, their milling succeeded in producing up to a 200 μm coating after 4 h. The use of light, scanning and transmission electron microscopy (LM/SEM/TEM) helped to establish that the coatings had gradient microstructures with more refined crystallites of NiAl, Al_3_Ni_2_ and CrB_2_ closer to the surface. With the addition of a ceramic phase, the coatings presented higher hardness and lower friction during dry wear tests both at RT and at 500 °C.

## 1. Introduction

Intermetallic coatings, due to their high hardness, provide an effective protection against wear for less resistant materials, like stainless steel [1,2,3,4]. Modifying them by the addition of ceramic particles enabled us to improve their properties even more [5,6,7]. The coatings serving this purpose should be characterized by high adhesion and thickness in the range from tens to hundredths of micrometers. The latter requirement helps to withstand the point mechanical loading, which is what protects coatings from local chipping. The presently available deposition techniques enable the production of coatings fulfilling both these requirements, but usually their other characteristics are less welcomed. It concerns especially a high porosity in those produced using atmospheric plasma spraying (APS) or even detonation gun (DG) techniques [8]. These defects make the coating permeable to the outside atmosphere, the exposure to which requires the introduction of an additional bond coat protecting substrates against oxidation during their high temperature (HT) cycling [9]. The above situation calls for the development of new ways of fabricating nano-crystalline composite coatings.

The cold-welding of metallic powder particles onto the balls and vial wall during mechanical synthesis of alloys originally was a nuisance and special processing control agents (PCA) were added to avoid or at least diminish its extent [10,11,12,13]. However, in recent years it started to be considered as a way to deposit coatings of various materials, including intermetallics, as well as composites [14,15,16]. The experiments performed with the pre-alloyed NiAl powders helped to establish that at the beginning of the deposition process the hard intermetallic particles were pressed in and welded into the softer substrates at a high rate. This rapidly progressing process was soon after that slowed down and stopped. This phenomenon was caused by changes taking place during ball milling of the powder, e.g., refined particles tended to form unyielding spherical agglomerates which pressed into the coatings that were being built and which were easily cracked and chipped off [17]. Therefore, it was practically impossible to cover the substrate with more than several tens of micrometers on average of the NiAl intermetallic or composite with such a matrix. It may seem a good achievement if it were not for the inherent high roughness of these coatings.

The NiAl intermetallic could also be mechanically synthesized from elemental Ni and Al powders [18,19,20,21]. The substitution of the pre-alloyed NiAl powders with the elemental ones during coatings deposition should affect especially the start of this process. It is the presence of softer metallic particles, like those of aluminum and nickel, which usually promotes a build-up of the deposit on balls and vial sides during the cold milling. With time, the ball milling will turn the elemental powders into the NiAl particles as well as refine the eventual addition of the CrB_2_ strengthening phase, allowing a return to the deposition of the NiAl or NiAl+ CrB_2_ coatings. Consequently, this effect should help to obtain thicker coatings over the substrates milled together with them. The coatings obtained in this way will present a gradient microstructure, this being a result of processes taking place during the high energy powder milling. The extent of each of the zones formed in the substrate and the material added over it during the milling of the Al and Ni powders with the admixture of CrB_2_ particles is of high significance as it concerns future application of these coatings.

Therefore, the present experiment aimed to investigate the possibility of deposition of NiAl+CrB_2_ coatings using co-milling elemental metal powders with ceramic particles and steel substrates. The microstructure and phase composition of the obtained coatings were investigated using the light, scanning and transmission electron microscopy (LM/SEM/TEM) methods, while micro-mechanical properties were assessed through friction tests and micro-hardness measurements.

## 2. Experimental Procedure

**Figure 1:**
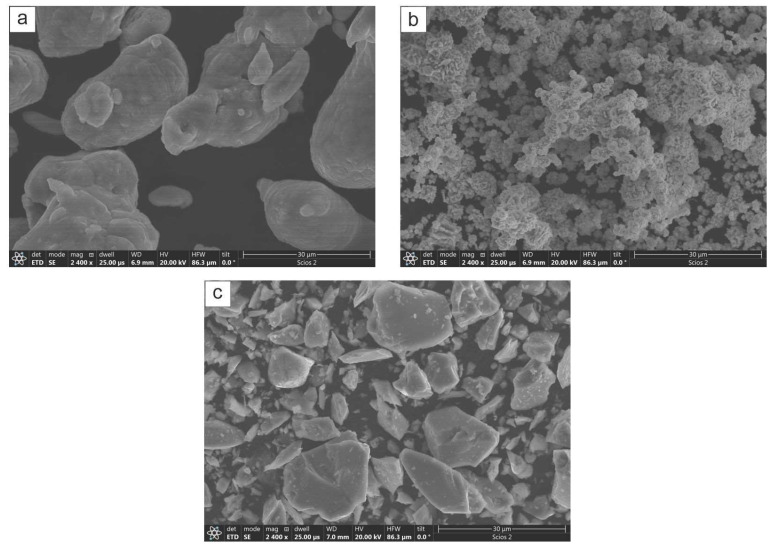
SEM images of as supplied powders of: (**a**) Al, (**b**) Ni and (**c**) CrB_2_.

The coatings deposition was performed in a planetary ball mill Pulverisette 5 (FRITSCH GmbH, Idar-Oberstein, Germany) using elemental powders of Ni (3N, av. size 45 μm, supplied by Alfa Asear, Haverhill, MA, USA) and Al (2N8, agglomerates av. size 40 μm, supplied by Alfa Asear) as well as CrB_2_ (2N, av. size 40 µm, supplied by Chem Ltd., Warsaw, Poland). The morphology of respective powders is shown in Figure 1. First, the Ni and Al powders were mixed at 1:1 molar ratio and next, 15 wt.% of CrB_2_ was added into it. Received powdery material was loaded into vials (of 0.5 L capacity) filled with steel balls (powder to ball ratio was kept at 1:10 ratio). The AISI 304 steel platelets (10 mm × 10 mm × 2 mm) were used as substrates. They were shot penned up to areal average roughness of S_a_ ~7 μm and co-milled with the powders. All steps involving powder mixing and loading into vials were performed in the glove box under a neutral argon atmosphere. The intermittent milling at 300 rpm with breaks of 45 min. every 15 min. of operation was applied with the aim of maintaining the temperature of processed materials at below 50 °C (temperature monitored with a K-type thermocouple). The deposition process was controlled by collecting one of the milled platelets after 4, 8, 16 and 32 h of deposition process. After taking off the vial, each one was treated in an ultrasonic cleaner for 10 min.

The micro-mechanical properties were measured with an FLC-50VX nano-indenter (Future Tech, Kawasaki, Kanagawa, Japan) under 0.5 N load (each time maintained for 15 s), while friction coefficient was determined with the UMT-2MT unit (CETR comp., Campbell City, CA, USA). The latter test was performed both at room temperature (RT) and at 500 °C using a corundum ball (diameter *ϕ*—3 mm) under 3N loading. Samples were sequentially mounted on a disc revolving at 0.1 m/s. The coating’s surface roughness and thickness were assessed using VHX-7000N digital light microscope (LM) (Keyence Corp., Itasca, IL, USA).

The microstructure and phase composition were characterized with the scanning and transmission electron microscopes (SEM/TEM), i.e., Scios 2 DualBeam (ThermoFisher Scientific, Eindhoven, The Netherlands) and Tecnai 200 kV FEG (FEI Company, Eindhoven, The Netherlands). The SEM imaging with back scattered electrons (BSE) was performed on metallographic sections of platelets ground on emery papers (up to 4000 grade) and finally polished using linen cloth pads sprayed with alumina suspension (grit 8000). The local chemical composition was measured with probe corrected Themis TEM (ThermoFisher Scientific, Eindhoven, The Netherlands) equipped with a large angle quad-silicon drift corrected EDS detector (Super-X). All mappings were built from a 500 × 500 pixel matrix. The lamellae for TEM observations were cut out with a beam of Ga+, i.e., focused ion beam (FIB) technique. The phase information was filtered by acquiring selected area electron diffraction patterns and solving them with the help of Process Diffraction software V_8.7.1 Q [22].

## 3. Results

**Figure 2:**
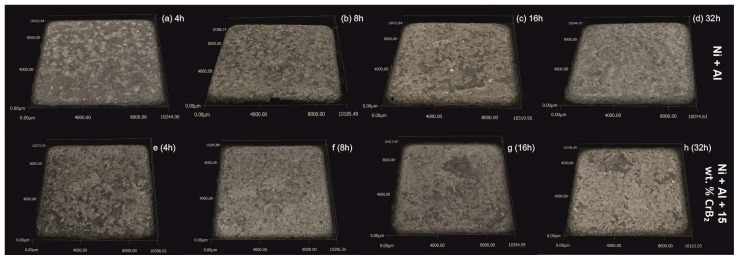
LM images of steel platelets milled with Ni+Al powders for: (**a**) 4 h, (**b**) 8, (**c**) 16 and (**d**) 32 h, and Ni+Al+CrB_2_ powders also milled for (**e**) 4 h, (**f**) 8, (**g**) 16 and (**h**) 32 h.

The qualitative assessment with the light microscope of the steel platelets milled together with elemental Al and Ni powders for 32 h showed that their surfaces bore numerous small dimples characteristic of materials after shot penning [23,24]. Otherwise, the platelets were fully covered with a greyish hue deposit, i.e., the color of the milled powders. After ultrasound cleaning in alcohol, their surfaces showed the presence of local luster areas corresponding to their stainless steel nature (Figure 2). The milling exceeding 8 h resulted in chipping away of larger areas of the deposited material, and was more frequent in the case of processes performed with the CrB_2_ addition. The above losses were rather limited to the upper part of the coating without exposure of the steel substrate.

**Figure 3:**
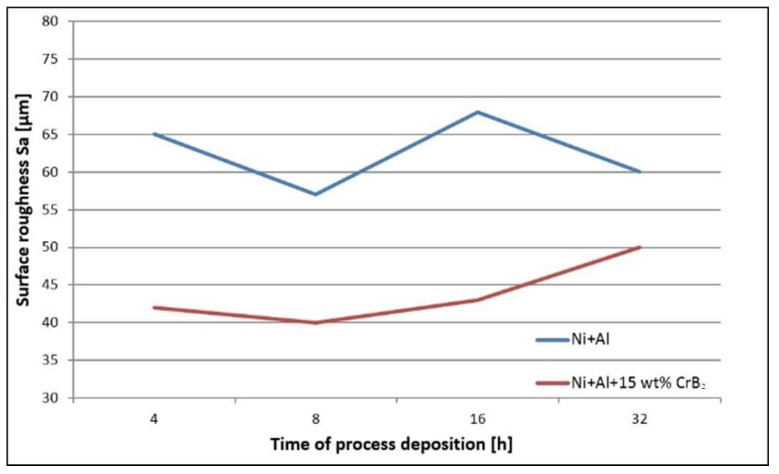
Graph representing changes in roughness of steel platelets with time milled both with Ni and Al and Ni, Al and CrB_2_ powders.

The quantitative assessment of the platelets’ topography helped to establish that the set milled with Ni and Al powders is characterized by ~40% higher roughness than the one milled with the addition of the ceramic phase, i.e., the average S_a_ was 40 μm and 60 μm, respectively (Figure 3). In the case of the former platelets, an extension of the milling time diminished the roughness by ~20%, but the latter platelet set, i.e., the one milled with the CrB_2_ addition, presented practically no changes within this processing time. The measurements performed for even longer times (16 h and 32 h) were taken from smaller areas free from chipping, but they were still useful to illustrate a tendency towards increased roughness with the milling time (plates milled with metallic powders for 32 h were the most affected by chipping).

**Figure 4:**
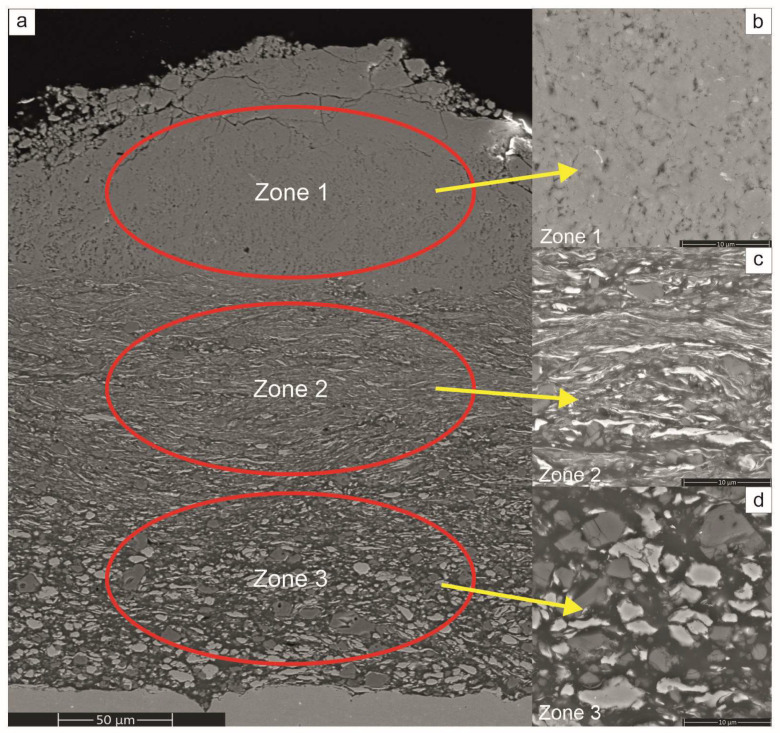
SEM overview image of section of Ni+Al+CrB_2_ coating obtained after 4 h of deposition process (**a**) showing its gradient microstructure, i.e., amorphous/fine crystalline (**b**), small flattened particles (**c**) and coarse blocky particles (**d**).

The SEM observations of sections of the platelets subjected to milling treatments showed that from the very early stages, i.e., even after 4 h of the processing time, they were all covered with a gradient coating with three distinct zones (Figure 4). The nearest to the substrate was built with predominantly a fraction of larger blocky particles, while the middle one was mostly occupied by flattened lamellar particles. The zone closest to the surface was filled with very fine particles relatively homogenously distributed in a featureless matrix. Comparing the coatings on the platelets milled with Ni, Al with Ni, Al and CrB_2_ powders showed similar microstructure evolution with time, although the process of reefing of ceramic particles was slower than it was for the metallic ones.

The gradient microstructure of the investigated coatings represents changes taking place during ball milling of metallic powders [10,25], i.e., they are fused together forming larger agglomerates; next, they are flattened forming corrugated strands while finally, they react, harden and break into nano-crystalline powder. Therefore, the coating formed by this mechanism has larger crystallites from starting powders built in near the substrate; above them, lamellar grains of Ni and Al are present and only the upper part of the coating is occupied by the intermetallic phases.

**Figure 5:**
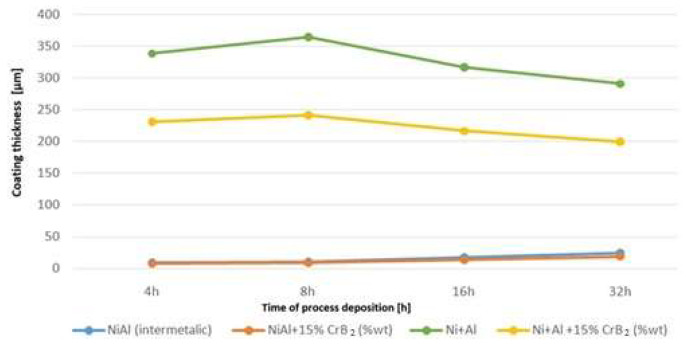
Graph representing deposition rate of coatings produced with Ni and Al powders, as well as with addition of CrB_2_ (for comparison, data concerning coatings obtained from milling steel platelets with pre-alloyed NiAl powder [17] were added).

**Figure 6:**
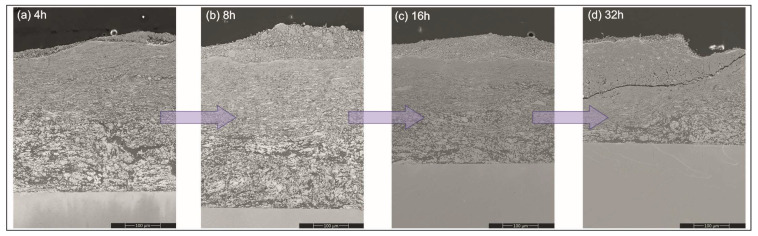
SEM images showing changes in coating microstructure during co-milling of platelets with Ni and Al powders for: (**a**) 4, (**b**) 8, (**c**) 16 and (**d**) 32 h.

Measurements of the coating’s thickness during the milling helped to establish that a significant amount of deposit from the milled materials was added up over the platelets already at the early stages of this process (Figure 5). Later on, it thickened at a slower rate, i.e., as the milling time was doubled from 4 to 8 h, coating thickness increased by ~5%. Longer processing times of up to 16 and 32 h resulted in an evident (~10%) diminishing of the amount of the added material. The cause responsible for it turned out to be a development of cracks nucleated at the surface and propagating slant wise until they hit the middle zone. Then, the cracks were deflected and continued along the boundary between them (Figure 6). The above process led to a removal of larger amounts of material from the near-surface zone causing a decrease in the coating’s overall thickness. Milling of the steel substrates with intermetallic NiAl powders gave a much thinner cover over them, but even after longer milling times it was an increase as documented in the previous experiment (see respective lines in Figure 5).

**Figure 7:**
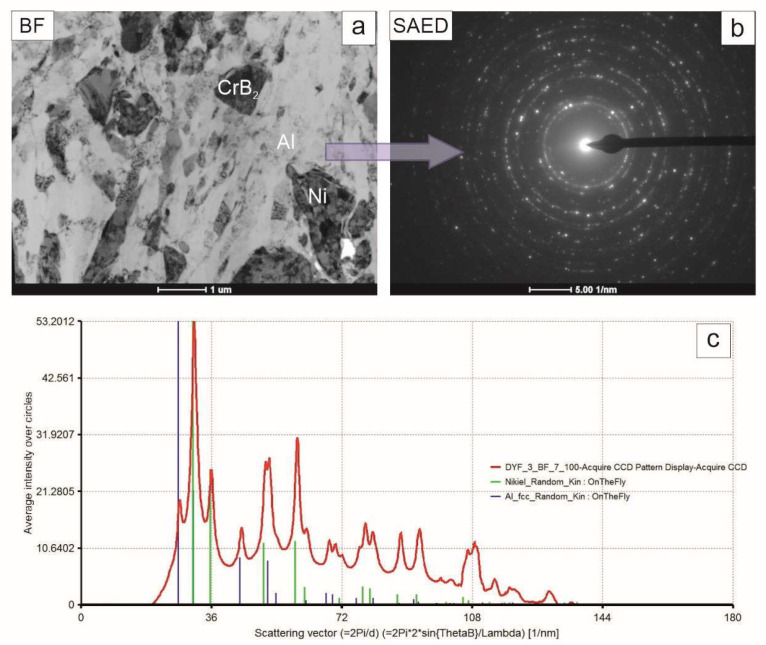
TEM image of coating near substrate zone (**a**), accompanying SAED pattern (**b**) and graph representing intensities of the latter integrated along radii (**c**) with markers corresponding to position of planes characteristic for nickel and aluminum.

The TEM observations of the coating near-substrate zone developed after 4 h of milling of Ni, Al and CrB_2_ powders showed that the blocky particles dominating it were either from the ceramic phase or were nickel grains tentatively identified basing on their different mass–thickness contrast (Figure 7). The matrix was formed from elongated aluminum grains with a minor admixture of similarly deformed nickel grains. The accompanying electron diffraction pattern acquired from that area upheld the assumption of the dominating presence of the aluminum and nickel phases in this area.

**Figure 8:**
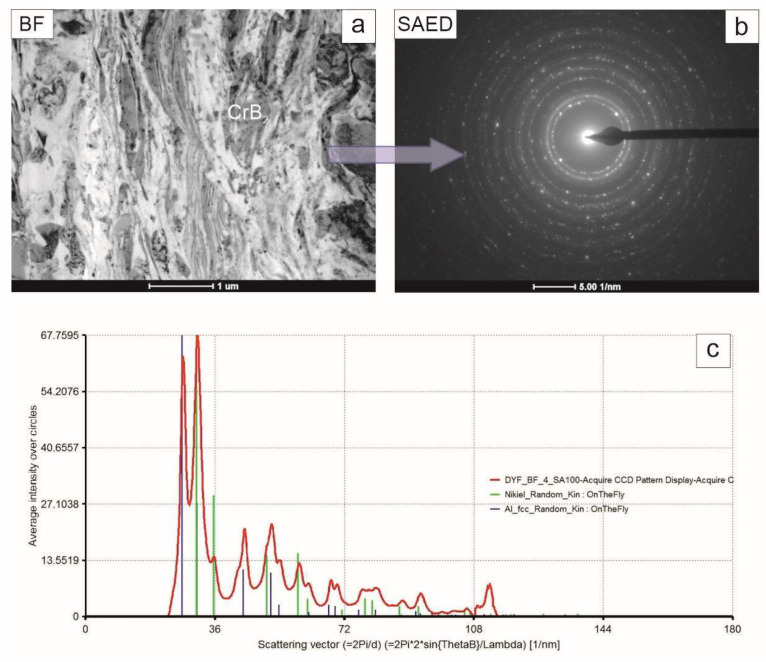
TEM image of coating middle zone (**a**), accompanying SAED pattern (**b**) and graph representing intensities of the latter integrated along radii (**c**) with markers corresponding to position of planes characteristic for nickel and aluminum.

The coating middle zone was built nearly exclusively from elongated grains of both nickel and aluminum phase with the occasional presence of CrB_2_, differentiating itself by its nearly defect free microstructure (Figure 8). The diffraction pattern acquired from that area consisted of rings with more numerous spots than in the previous case, reflecting its refined microstructure. The summed intensity in the attached graph again allowed us to identify only nickel and aluminum.

**Figure 9:**
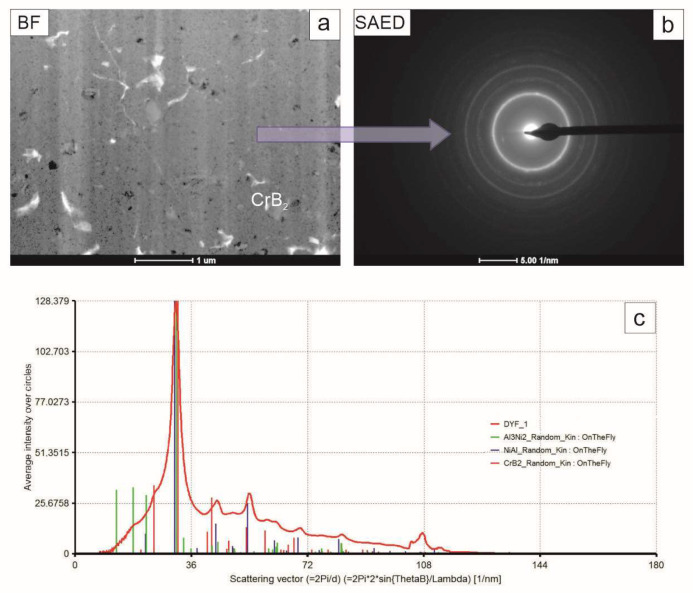
TEM image of coating near-surface zone (**a**), accompanying SAED pattern (**b**) and graph representing intensities of the latter integrated along radii (**c**) with markers corresponding to position of planes characteristic NiAl, Al_3_Ni_2_ and CrB_2_ phases.

The coating outer (near-surface) zone was the most uniform one (Figure 9). It was characterized by the presence of very small crystallites of CrB_2_ phase in an amorphous-like matrix. The electron diffraction patterns acquired from that area consisted both of diffused and sharp rings, confirming the presence of amorphous and fine crystalline material. This time, the summed intensity of the rings showed peaks at positions proper for the NiAl and Al_3_Ni_2_ intermetallics, as well as the CrB_2_ compound. The lack of the latter phase in the patterns obtained from the zones below was caused by the fact that the ceramic particles located over there were of much larger size and were much less frequent. Consequently, as diffraction was acquired from the most representative areas, i.e., those free of larger particles, this signal was skipped. In the near surface area, CrB_2_ particles were so refined that quite a number of them were always involved in diffracting the electron beam. The diffused intensity in between the rings confirms the substantial share of amorphous material. This partly crystalline and partly amorphous matrix was filled with cracks, which were frequently deflected at CrB_2_ particles.

**Figure 10:**
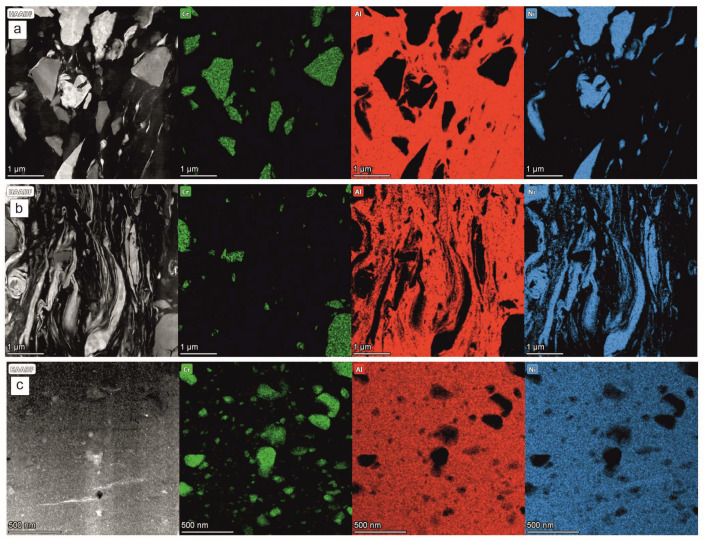
STEM/HAADF images of coating on near-substrate (**a**), middle (**b**) and near-surface (**c**) zones, accompanied by maps showing distribution of Cr, Al and Ni elements obtained after 4 h of milling.

The TEM/EDS maps showing the local chemical composition of the coating formed after 4 h of milling helped to positively verify the information obtained from the TEM/BF observations and the SAED measurements: within the near-substrate zone in between the Al and Ni grains there was no inter-diffusion (Figure 10a). They also showed that even if most of the CrB_2_ crystallites (represented by Cr rich areas) preserved their shape, some were cracked. In effect, the presence of numerous, very small CrB_2_ particles was noted. Interestingly, the same situation seemed to exist in the middle zone, i.e., the refining and flattening of the particles also took place without the inter-diffusion processes (Figure 10b). However, it completely changed in the case of the near-surface zone, where the metallic matrix presented a homogenous distribution of the nickel and aluminum (Figure 10c). The size of the largest CrB_2_ particles decreased by two to four times, and their shape was rounded in the milling process. The matrix was also filled with numerous much finer particles of the same compound, which was probably a result both of chipping of the larger ones and the total shattering of the rest.

**Figure 11:**
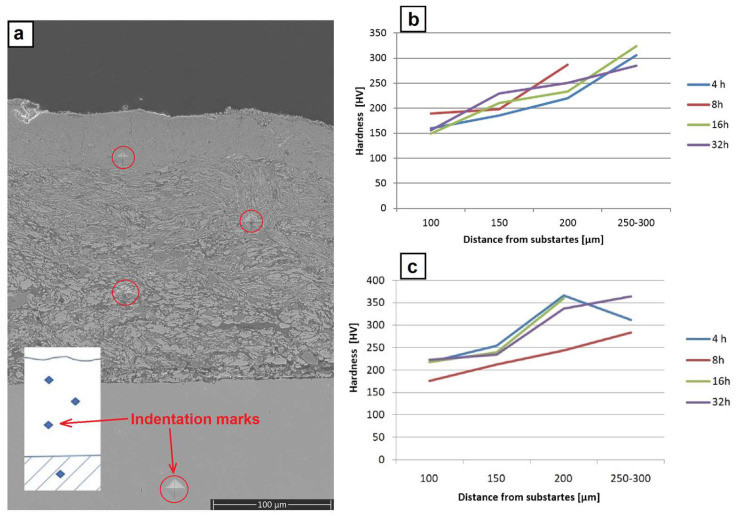
SEM image of section of coating over platelets milled with Ni, Al and CrB_2_ with marking left after indentation (**a**), graph representing hardness changes with distance from substrates milled with Ni and Al powders (**b**) as well as in Al, Ni and CrB_2_ (**c**).

The hardness measurements were performed at the polished sections of the coatings, as shown in Figure 11a. On the one obtained through the milling of the platelets with metallic powders, they showed a gradual increase from ~160 HV close to the substrate, up to ~220 HV in the middle of the coating and up to ~300 HV close to the surface (Figure 11b). The highest scatter of hardness during these measurements was documented for an indent located in the middle of the coating, which might have been caused by the fact that this area was filled with intermixed highly flattened particles strongly differing in thickness. The same measurements performed for the coating formed through the milling with metallic powders containing CrB_2_ ceramic admixture showed significantly higher values in all specified areas, i.e., ~220 HV, ~300 HV and ~350 HV, respectively (Figure 10c). In this case, the scatter of averaged hardness values was the highest for the near surface areas for the coating obtained after 4 h of milling. It was caused to a large extent by the fact that after so short a processing time the matrix was already nano-crystalline and highly brittle, while some of the CrB_2_ particles were still imbedded in it, promoting cracks and local chipping of the freshly deposited material.

**Figure 12:**
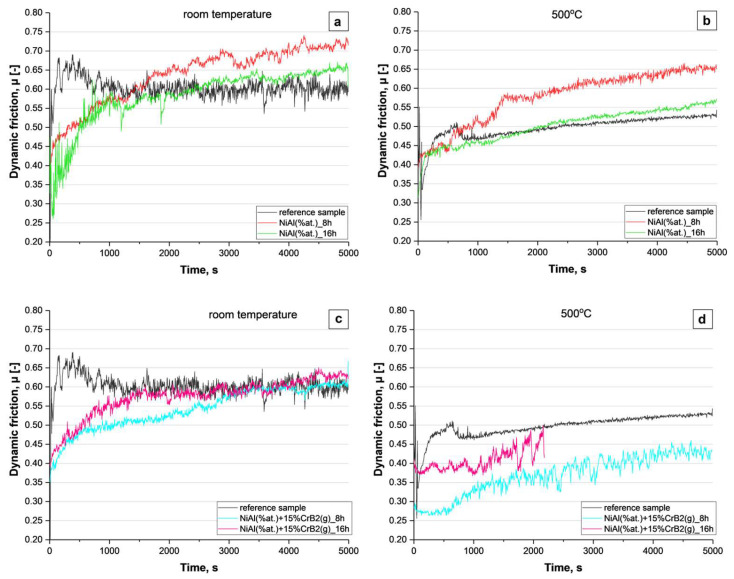
Graphs representing changes in friction during ball-on-disc test performed at RT and 500 °C for coatings produced with metallic powders (**a**,**b**) and with addition of CrB_2_ (**c**,**d**).

The wear test performed at room temperature, as well as at 500 °C, on platelets milled with Ni and Al powders for 8 and 16 h showed that it is only at the beginning that the friction is lower than that presented by reference steel (Figure 12a,b). Later on, it was on the same level or even slightly higher than the latter. It means that the coatings produced from metallic components wear down very fast (like stainless steel) and in later stages this process was additionally accelerated by hard debris (friction being higher than in the case of the reference material). The test involving coatings deposited with Ni, Al and CrB_2_ powders indicated that especially the ones milled for a shorter time (8 h) presented a lower level of friction for a significantly longer time (Figure 12c). It means that the addition of a ceramic phase effectively prevented its premature removal. It is worth noting that this coating, subjected to wear at 500 °C, had withstood the test with a friction coefficient still lower than that presented by the reference material, e.g., steel (Figure 12d). The longer milling times, i.e., 16 h, however, caused a deterioration of the resistance to degradation by abrasion.

**Figure 13:**
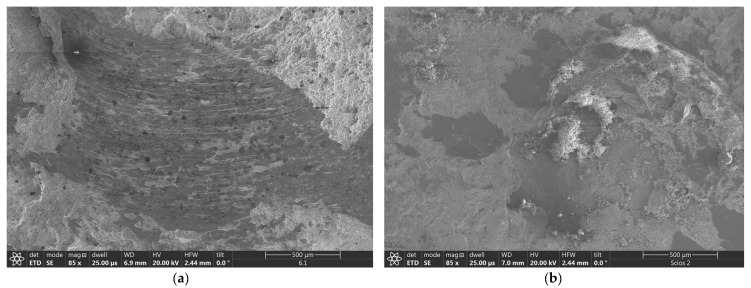
SEM images showing wear track formed during HT test (500 °C) on: (**a**) NiAl and (**b**) NiAl+CrB_2_ coatings deposited on steel substrates after 8 h milling time.

The SEM observations of the wear tracks over NiAl coatings showed that the friction of the corundum ball caused local removal of larger parts of coatings producing on its way a string of abrasion grooves (Figure 13a). The same test with NiAl+CrB_2_ coatings formed evidently shallower scars after the same length of time. Additionally, wear tracks over these coatings were accumulating more wear debris, producing a characteristic charging effect during SEM observations (Figure 13b).

**Figure 14:**
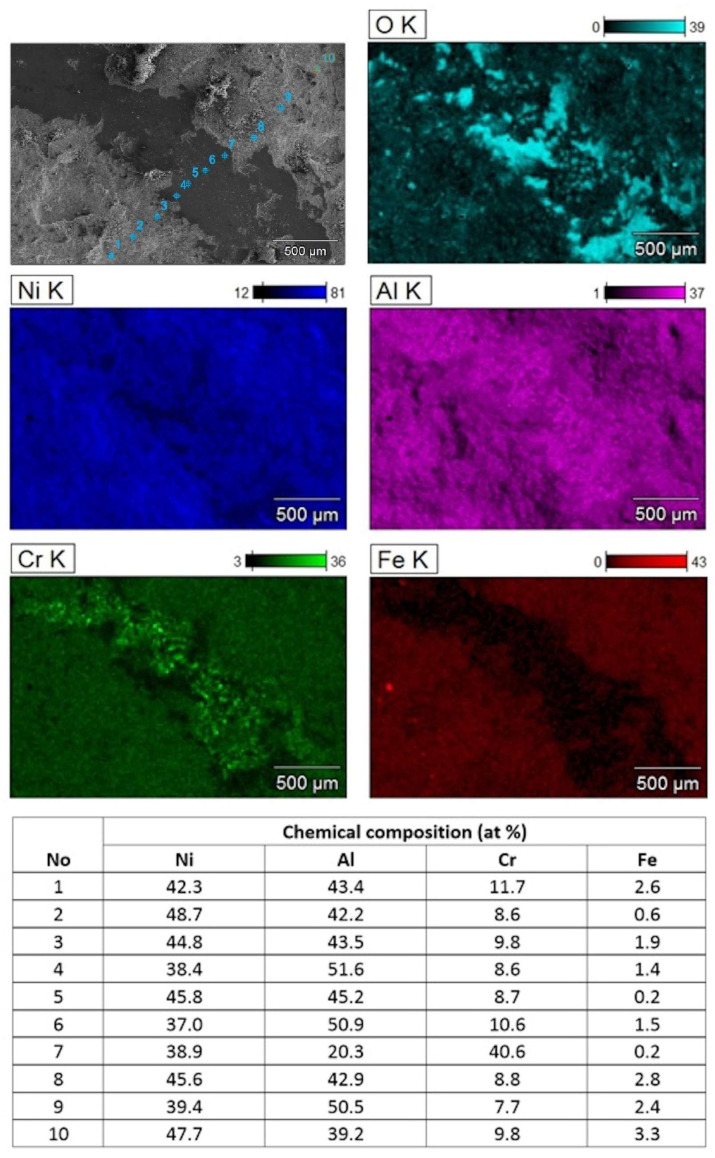
SEM image of one of deeper depressions in wear track formed during HT test (500 °C) on NiAl+CrB_2_ coatings deposited on steel substrates after 8 h milling time and accompanying maps showing distribution of Al, Cr, Fe, Ni and O. Table below presents chemical compositions measured across wear track (B and O were omitted as unreliable in case of rough surfaces) at points marked on SEM image.

The SEM/EDS mapping of the local chemical composition from the bottom of deeper parts of wear tracks formed on the NiAl+CrB_2_ coating confirmed that it was still formed mostly from the material rich in nickel and aluminum and covered with heaps of oxidized wear debris (Figure 14). However, the presence of distinct signals from CrB_2_ particles at the track bottom means that the near surface layer formed from amorphous/nano-crystalline NiAl and CrB_2_ crystallites was penetrated. Otherwise, the chromium signal from that zone would have been intermixed with the Ni and Al like it was in neighboring areas at the outside of the wear track. The iron particles documented on the map showing the distribution of Fe must have, most probably, got over there from the vial walls, balls or substrates (all of them are made from steel). The measurements of the relative content of the Ni and Al at the sides of wear tracks confirmed that at the near surface areas their shares are usually close to 1:1, while at the wear track bottom, local enrichment in Al is more frequent.

## 4. Discussion

The co-milling of elemental Al and Ni powders with the steel platelets (serving as substrates) in the planetary ball mill allowed us to produce coatings of up to a few tens of millimeters thickness. The intermetallic coatings of thicknesses in that range could also be formed using industrially established techniques like atmospheric plasma spraying (APS), high velocity APS (HV-APS) [2] or detonation gun [9]. Additionally, their thickness could be tailored to specific needs within a large margin, while their surface roughness is usually kept below 20 μm [26]. The present approach is much less flexible as after reaching a certain surface hardness, the chipping of pieces of the coatings prevails over the addition of new material. What is more, the roughness of these coatings exceeds by far that achieved for PSA or GD ones. Therefore, an advantage of the coating formed by mechanical embedding of powder particles should rather be sought in applications in which deposit, during the mechanical synthesis of materials, is readily accumulating on, for example, the vial walls. The inner sides of short cylinder sections resembling the latter are simultaneously relatively hard to coat with the APS, HV-APS or DG techniques.

All obtained coatings showed a gradient microstructure on which successive zones were filled with a material preserved from respective stages of milling the elemental powders. Starting from the substrate, one may find in them heavily dislocated but single- phase grains of Ni and Al which are substituted with flattened packets of strung out grains and it is only near the surface that a layer of nano-crystalline/amorphous material is added. Such a situation is caused by the fact that in the mechanical embedding process, the newly added material shields the one below. In effect, the microstructure of subsequent layers of the coatings deposited in that way corresponds to microstructure evolution during ball milling of metallic elemental powders [10]. It should be noted that within this gradient coating, the NiAl and Al_3_Ni_2_ intermetallic phases immersed in the amorphous NiAl matrix occupy only its upper part. However, this zone is still of a significant thickness of several tens of micrometers and of unique glassy matrix/nano-crystalline composite structure [27], being hard to reproduce with any other deposition technique.

The dry wear test at room temperature (RT) performed with NiAl coatings or that involving the addition of CrB_2_ particles indicated that their performance, as represented by dynamic friction, is practically on a par with a reference steel sample. The same test repeated at high temperature (HT) again showed only mediocre results for NiAl coatings, but very good results for NiAl+CrB_2_. It should be noted that at 500 °C (present wear test temperature), the amorphous NiAl, forming a matrix on the upper part of the investigated coatings, softens up. It is because this temperature exceeds the glass transition temperature Tg (475 °C) of this alloy [28]. The malleable matrix with well dispersed hard TiB_2_ particles replicates the microstructure of Babbitt alloys developed for sliding bearings [29,30], i.e., a soft tin matrix with load carrying Sb_2_Sn_3_ precipitates. Even in the case where this hypothesis would hold, the use of the present coatings for wear protection would be of limited use as the glass transition range for NiAl covers only several tens of centigrade. Therefore, these coatings should be rather considered as a protection against corrosion than wear. In such cases, the presence of Al_3_Ni_2_ aside from NiAl would be of advantage as its oxidation is more prone to form a dense alumina coating stopping diffusion and protecting the material below [31]. The amount of Al_3_Ni_2_ phase in these coatings could be easily diminished by changing the Al:Ni ratio in the starting powder, but the present setting was helpful in building up the thicker deposit.

## 5. Summary and Conclusions

The co-milling of stainless steel (ASI 304) platelets with elemental Ni and Al powders eventually allowed us to cover them with a much thicker coating than that obtained with pre-alloyed NiAl powders. The microstructure investigation and micro-mechanical test allowed us to state that:

- The first hours of milling turned out to be the most effective in imbedding powder particles over the platelets, while during their longer processing, chipping of the brittle parts from the coating surface was prevailing over the deposition of the new material.
- The coating was characterized by a gradient microstructure consisting of three zones, i.e., the nearest to the substrate, built of blocky Ni and Al grains, and the middle one, occupied by refined and flattened Ni and Al grains, while the zone closest to the surface was filled mostly with nano-crystallites of the NiAl and Al_3_Ni_2_ intermetallic phases immersed in an amorphous NiAl matrix.
- Adding into the milled metallic elemental Ni and Al powders an amount of up to 15 wt.% of CrB_2_ ceramic phase allowed us to obtain composite coatings of a gradually decreasing size of borides immersed in the matrix of the same gradient microstructure as described above.
- The significant improvement of the dry wear resistance was achieved only for the NiAl+CrB_2_ coating tested at 500 °C. This may have its explanation in the softening of the amorphous NiAl matrix strengthened with ceramic particles, i.e., conditions proper for operation of the mechanism being used in the case of Babbitt alloy used in sliding bearings.

## Data Availability

Data are contained within the article.
